# Enantioselectivity and residue analysis of cycloxaprid and its metabolite in the pile and fermentation processing of Puer tea by ultraperformance liquid chromatography–high‐resolution mass spectrometry

**DOI:** 10.1002/fsn3.2901

**Published:** 2022-04-22

**Authors:** Hongcheng Liu, Hen Tian, MingMing Jiang, Tao Lin, Ji Li, Xiangzhong Huang

**Affiliations:** ^1^ Institute of Quality Standard and Testing Technology Yunnan Academy of Agricultural Science, Supervision and Testing Center for Farm Product Quality Ministry of Agriculture Kunming China; ^2^ Key Laboratory of Chemistry in Ethnic Medicinal Resources Yunnan Minzu University Kunming China; ^3^ School of Pharmaceutical Science & Yunnan Key Laboratory of Pharmacology for Natural Products Kunming Medical University Kunming China

**Keywords:** cycloxaprid, enantioselectivity, metaboliteLC‐HFMS, puer tea processing

## Abstract

The residues of cycloxaprid enantiomers and metabolites are investigated by ultraperformance liquid chromatography–high‐resolution mass spectrometry (UPLC‐HRMS) during raw and ripen Puer tea processing. A Chiralpak AG column with chiral stationary phase of amylose tris (3‐chloro‐5‐methylphenylcarbamate) is succeed to separate the *1R, 2S*‐cycloxaprid, *1S, 2R*‐cycloxaprid, and their metabolite, which is identified as nitrylene‐imidazolidine. It is not conversed *1R, 2S* **‐**cycloxaprid into *1S,2R*‐cycloxaprid during Puer tea processing. The estimated half‐lives of the *1R,2S*‐cycloxaprid and *1S,2R*‐cycloxaprid are 0.97 and 1.1 h, respectively, and *1R,2S*‐cycloxaprid decreases more quickly than the *1S,2R*‐cycloxaprid. During raw Puer tea processing, the half‐lives of *1R, 2S*‐cycloxaprid and *1S, 2R*‐cycloxaprid are 1.68 h and 1.77 h, but the residue is still detected even if it is over 730 day. However, the half‐lives of *1R,2S* **‐**cycloxaprid and *1S,2R*‐cycloxaprid are 0.60 day and 0.63 day during ripen tea processing. The amounts of metabolite are more in raw tea than in ripen tea; the terminal residues are still detected until 730 days during raw tea. A significant enantioselectivity of *1R, 2S*‐cycloxaprid and *1S, 2R*‐cycloxaprid is observed during raw tea or ripen tea processing. The degration result shows the enantioselectivity of cycloxaprid in raw or ripen Puer tea processing.

## Graphical abstract

The metabolite, which is identified as 1‐(6‐chloronicotinyl)‐2‐nitromethylene‐imidazolidine.The metabolite of cycloxaprid in raw or ripen Puer tea processing.

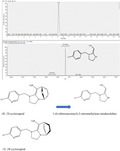

## 1 INTRODUCTION

**FIGURE 1:**
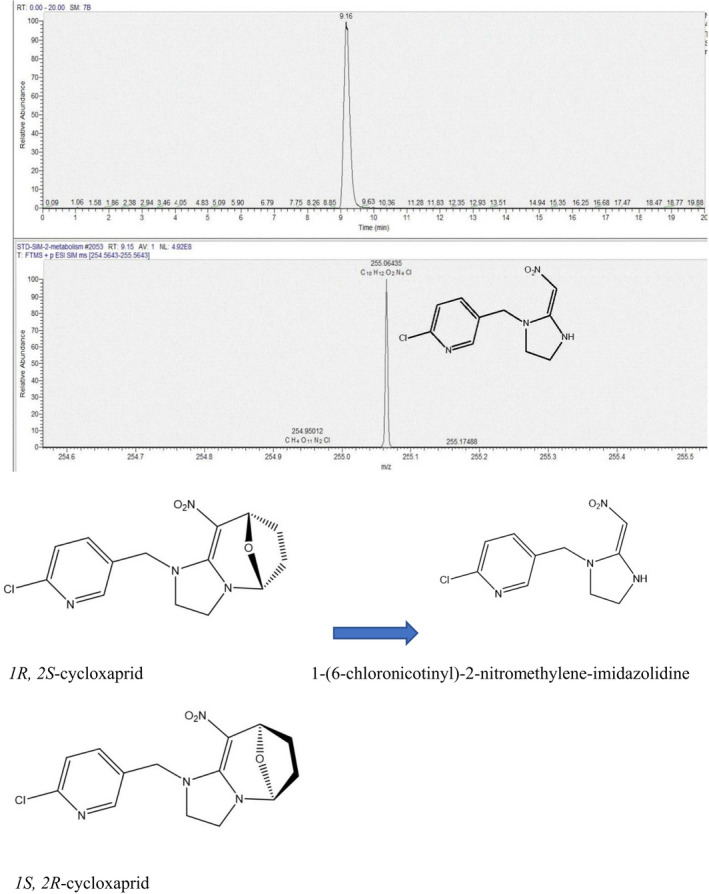
typical LC‐HRMS chromatograms of metabolite and the transformation of cycloxaprid

Neonicotinoids are the most important class of synthetic insecticides for tea protection against piercing‐sucking pests (Tomizawa & Casida, 2003). Cycloxaprid is a new neonicotinoid insecticide that has been synthesized and industrialized in China (Li et al., 2011). It is different from traditional neonicotinoids, which act as agonists of native and recombinant nicotinic acetylcholine receptors (nAChRs ) (Liu & Casida, 1993; Matsuda et al., 1998; Nishimura et al., 1994; Tomizawa & Casida, 2003) and show high insecticidal activity against a broad spectrum of sucking and biting insects1 (Cui et al., 2012; Shao et al., 2010), which suggests that it has been considerable as the third generation of neonicotinoids. The molecular structure of cycloxaprid contains a chiral oxabridged cis‐configuration leading to a pair of enantiomers, *1R,2S*‐cycloxaprid and *1S,2R*‐cycloxaprid (Figure 1). Cycloxaprids are commonly produced and used as racemic mixtures and the stereoselectivity degrade is found in soils (Liu et al., 2015). Zhang et al. observed stereoselective uptake and translocation of cycloxaprid in edible vegetables from roots (Zhang et al., 2013). However, Chen et al. (Chen et al., 2017) founded adverse results, as evidenced by the lack of significant difference between the stereoisomers in their fate in aerobic soils and three mainly metabolites were found in soil.

The metabolite of cycloxaprid is readily founded through photolysis, hydrolysis, and oxidation reaction. Liu et al. (Liu et al., 2015) identified and tracked 11 metabolites of cycloxaprid, and tracked their changes in flooded and anoxic soils. Shuang et al. (Hou et al., 2017) studied the photostability of cycloxaprid in water and detected 25 photodegradation products; the predominant photodegradation product was named as NTN 32,692. Fang et al. (Fang et al., 2017) reported that the degradation dynamics of two neonicotinoids during *Lonicera* japonica planting, drying, and tea brewing processes were researched. Hou et al. (Hou et al., 2013) compared the dissipation behavior of three neonicotinoid insecticides in tea and found high transfer rates through green or black tea brewing of 80.5% or 81.6% for thiamethoxam, of 63.1% or 62.2% for imidacloprid, and of 78.3% or 80.6% for acetamiprid. However, the degradation behavior and metabolite of cycloxaprid was still unknown in Puer tea processing.

Liquid chromatography–high‐resolution mass spectrometry (LC‐HRMS) has also been explored and has shown great potential for untargeted profiling in tea (Gao et al., 2019; Jia et al., 2020). The analysis method of cycloxaprid in tea was scarcely by LC‐HRMS. Only Liu et. al. (Liu & Jiang, 2020) reported that the stereoisomer behavior of sulfoxaflor was determined by LC‐HRMS during Puer tea and Black tea processing.

Therefore, a new analytical method is developed to determine stereoisomer of cycloxaprid and metabolite in Puer tea using LC‐HRMS. The method was applied to investigate the stereoselective cycloxaprid degradation during Pu‐erh tea processing.

## 2 MATERIALS AND METHODS

### 2.1 Chemical materials

A racemic mixture of cycloxaprid was provided by Beilinwei technology Ltd. Cycloxaprid powder 25% was supplied by Shanghai Shengnong pesticide Co Ltd. Ten milligram of 2‐chloro‐5‐[[−2‐(nitromethylidene) imidazodin‐1‐yl] methyl] pyridine (CAS 10336–63–4) was synthesized by Alta Scientific Co., Ltd. The enantiomerically pure standards: *1R,2S*‐cycloxaprid, *1S,2R*‐cycloxaprid were separated by semipreparative HPLC with Chiralpak AG, polysaccharide‐based chiral stationary phase consisting of amylose tris (3‐chloro‐5‐methylphenylcarbamate), 250 × 4.6 mm i.d., 5 μm.

The stock solutions were produced by dissolving the cycloxaprid in acetonitrile. All solutions were stored in a refrigerator at ‐ 18°C. HPLC‐grade acetonitrile and methanol were provided by Tedia Company Inc. The initial dose of cycloxaprid 50 mg/L was used with 1g of cycloxaprid completely dissolved into 5 L water. Water was purified using a Milli‐Q system.

### 2.2 Separation the metabolite of cycloxaprid

One gram of 25% cycloxaprid powder is placed on sun at 3 d. The sample is dissolved by 20 ml water, then extracted by acetonitrile. The metabolite with degradation test is obtained by semipreparative HPLC. The molecular structure of metabolite is analyzed by LC‐HFMS.

### 2.3 Separation optical pure standards:*1R,2S*‐cycloxaprid and*1S,2R*‐cycloxaprid

The sample of 10 mg/ml cycloxaprid is separated by semipreparative HPLC. The stereoisomer of cycloxaprid was separated by using a Chiralpak AG (amylose tris (3‐chloro‐5‐methylphenylcarbamate) as stationary phase, 250 × 4.6 mm i.d., 5 μm, Daicel Ltd. JP). Mobile phases are H_2_O and acetonitrile (55:45), respectively. The flow rate was 1.0 ml/min. the injection volume is 40 μL.

### 2.4 The transform of optical pure compounds in Puer tea processing

The optical pure standards of *1R,2S* **‐**cycloxaprid or *1S,2R*‐cycloxaprid (1 mg/L) are respectively added to research the transform of optical pure compounds in Puer tea processing. The test at intervals time is designed at 0, 2 h, 15 h, 24 h, 48 h, 96 h, and 140 h.

### 2.5 Degradation in raw Puer tea

Sun‐dry Puer tea (20 kg) is obtained and sprayed with 50 mg/L aqueous solution (25% powder) in March, 2019. The raw Puer tea is stored under air temperature (5–28°C) and in dark place. The intervals time is designed at 0 (2 h), 4 h, 10 h, 16 h, 1d, 3d, 6 months, 12 months, 18 months, 24 months, and 36 months. The sample is dried to constant weight and the residues amount calculated with dry sample.

### 2.6 Ripen Puer tea processing

To ferment the ripen Puer tea, 20 kg of sun‐dry Puer teas is sprayed with 25% powder at the dose of 50 mg/L aqueous solution to keep the conditions of 35% moisture content. During the pile‐fermentation, the fermented tea was artificially turned and piled again at 7th days (first pile), 14th days (second pile), the facial pile‐fermentation tea is splashed by 100 ml water at 3 day intervals. The sample is collected at intervals time on 0 (2 h), 1, 3, 7, 15, 30, and 45 days. The residues amount expressed as dry sample.

### 2.7 Calculation of enantiomer fraction

The enantiomer fraction (EF) was $\text{EF}=\frac{R}{\left(R+S\right)}$, concentration of the *1R,2S*‐cycloxaprid was R, the other concentration of the *1S, 2R*‐cycloxaprid was S.

### 2.8 Samples preparation

Five grams of sample was exactly weighed and added 10 ml water, 20 ml acetonitrile. After the mixture was vortexed, 5 g NaCl was added. The tube was shaken vigorously for 1 min using a vortex mixer and centrifuged at 5000 rpm for 5 min. The upper layer solution was mixed with 150 mg PSA and 150 mg anhydrous MgSO_4_ for cleanup. After shaking and centrifugation at 5000 rpm for 3 min, 0.5 ml of the upper layer was filtered through 0.22 μm filter for LC‐HFMS analysis.

### 2.9 UPLC‐HRMS Analysis

Sample analysis was achieved in an ultraperformance liquid chromatography–Q exactive high‐resolution mass spectrometry (Thermo Fisher Scientific,) system.

The instrument was tuned in the positive ESI mode (3.8 kV of spray voltage, 325°C of capillary temperature, 350°C of probe heater temperature, and 60 V of SLens). The instrument was calibrated using positive calibration solutions every three days. The pesticide analysis was performed in FS/DIA mode. In FS, the scan range was set at *m/z* 150–750; mass resolution at 140,000 FWHM; AGC target and maximum IT were set at 1.0 e6 and 100 ms, respectively. For DIA, the relevant parameters were set as follows: mass resolution: 140,000 FWHM; AGC target: 2 e^5^; maximum IT: 30 ms; Loop count: 12; MSX count: 1; Isolation window: 50 Da; stepped normalized collision energy (NCE): 20%, 40%, and 60%. The spray voltage in positive and negative modes was set as 3.5 kV and 3.0 kV, respectively. And in our experiment, the positive and negative modes were analyzed separately. The flow rate of sheath gas and aux gas was 45 and 10 (in arbitrary units), respectively.

### 2.10 Method validation

The method was validated with the following parameters: matrix effect, accuracy, linear range, limit of detection (LOD), limit of quantification (LOQ), specificity, and precision. The standard solution was determined from 2.0 to 100 μg/ml concentration for each enantiomer. Three times signal‐to‐noise (S/N) ratio was as the LOD for every enantiomer, whereas the LOQ was based on the lowest spiked concentration level. As shown in Table 1, standard solution calibration curves were good in the range of 0.2–50 μg/L for *1R,2S*‐cycloxaprid, *1S, 2R*‐cycloxaprid. Linearity was satisfactory with mean correlation coefficients (R2) >0.99.

**TABLE 1:** Limits of detection, quantification, calibration equation

	LOD (µg /kg)	LOQ (µg /kg)	Calibration equation (*n* = 5)	Determination coefficient, *R*2	Linear range tested (µg/L)
*1R,2S*‐cycloxaprid	0.2	1	y = 62137x +7289	0.998	0.2–50
*1S,2R*‐cycloxaprid	0.2	1	y = 24244x −2547	0.999	0.2–50
2‐Chloro−5‐[[−2‐(nitromethylidene) imidazodin−1‐yl] methyl] pyridine	0.1	1	y = 34769x +1781	0.998	0.05–10

The result of recoveries (1 and 10 µg/kg) and the precision (RSD) was seen in Table 2. The mean recoveries were good from 72.4% to 92.7%, and the RSD were below 10% at the three fortified levels in tea samples. The results were shown that the accuracy and precision was guaranteed for enantiomeric analysis.

**TABLE 2:** The recovery and RSD of an analyses spiked in Puer tea at two concentration (*n* = 6)

Enantiomer	Addition (µg/kg)	Raw Puer tea		Ripen Puer tea	
		Recovery %	RSD %	Recovery %	RSD %
*1R, 2S*‐cycloxaprid	10	93.2	6.8	94.7	6.2
	1	88.4	2.8	84.6	4.6
*1S, 2R*‐cycloxaprid	10	90.6	8.6	98.2	3.6
	1	85.2	5.7	95.1	4.7
2‐Chloro−5‐[[−2‐(nitromethylidene) imidazodin−1‐yl] methyl] pyridine	10	88.7	3.8	86.3	5.5
	1	84.9	4.1	82.5	3.9

## 3 RESULTS AND DISCUSSION

### 3.1 Separation and Identification of metabolite

Because the cycloxaprid contain oxabridged structure, it is unstable under air temperature. To obtain the transformation, the conversion of cycloxaprid is quickly generated by sun light. During sun light, one new compound are detected and separated by HPLC at third test. Tentative structural identification of the transformation products was made by LC‐HFMS. The mass spectrum *m/z* were 255.06435 [M + H]^+^; the chemical structure was C_10_H_12_N_4_O_2_Cl, Delta ppm 0.079. Chen et al 2017 (Chen et al., 2017) reported the same mass spectrum with *m/z* 255.0645 [M + H]^+^ and 255.06 [M + H]^+^ (Hou et al., 2017) the chemical structure of metabolite was identified by artifical synthesis by Alta Scientific Co., Ltd. The metabolite and standard were simultaneously analyzed by LC‐HFMS, and their retention time, molecular ion peak, and fragmentation pattern were in agreement with the synthesized structure standard. So the metabolite was unambiguously identified as 2‐chloro‐5‐[[−2‐(nitromethylidene) imidazodin‐1‐yl] methyl] pyridine (CAS 10336–63–4) (NMI). (Figure 1).

### 3.2 Chromatography Separation optimized

Because of absence of oxabridged ring, the metabolite was unstereoselective molecule. Once cleavage occurred on the oxabridge, the metabolite is no longer enantioselectivity. To simultaneously separate the chiral cycloxarpid and metabolite, the reverse‐phase chiral columns were employed which contained cellulose‐ and amylose‐based polysaccharide materials; a cellulose‐based column (Chiral Cel OJ‐3R) and two amylose‐based columns (Chiralpak AD‐RH and Chiralpak IG) were tested using a variety of reverse‐phase mobile phase combinations.

Racemic cycloxaprid could not be resolved using methanol/water or acetonitrile/water by Chiralcel OJ‐3R; however, enantio‐separation was well obtained by two amylose‐based columns (Chiralpak AD‐RH and Chiralpak IG). The results showed that enantiomer cycloxaprid was more easily resolved in amylose‐based columns than in cellulose‐based columns. The Chiralpak IG column contained a new generation of polysaccharide‐based amylose tris(3‐chloro‐5‐methylphenylcarbamate) and showed the best enantiomeric resolving ability for enantiomer cycloxaprid and metabolite using a mixture of ACN/water (v/v: 35/65) as the mobile phase, flowing rate with 0.6 ml/min.

### 3.3 The transformation and degradation of optical pure compounds

*1R,2S* **‐**cycloxaprid and *1S,2R*‐cycloxaprid (1 mg/L) is separated by chiral column Chiralpak AG 250 × 4.6 mm i.d., 5 μm) with semipreparative HPLC.

**FIGURE 2:**
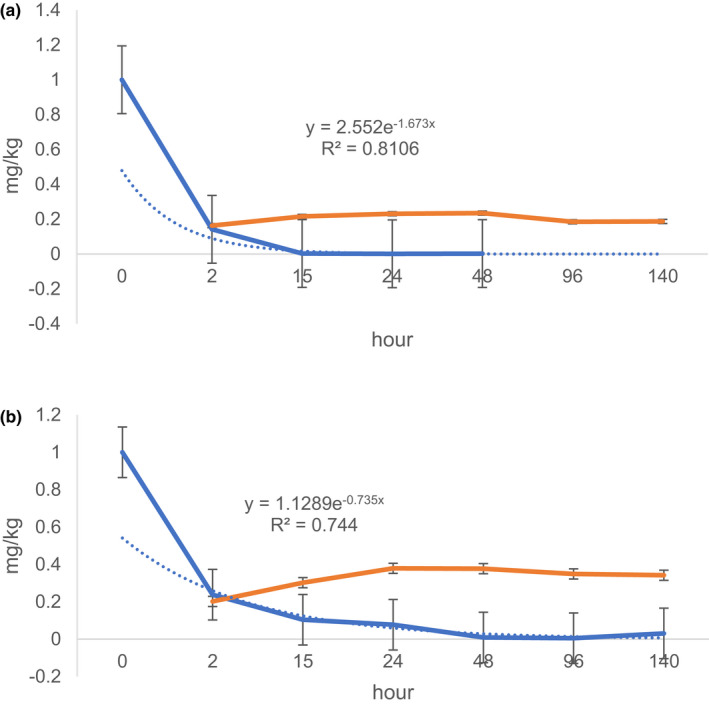
Dissipation kinetic of optical pure compounds (a) *1R, 2S*‐cycloxaprid, (b) *1S, 2R*‐cycloxaprid and increase of metabolite

The transformation test of *1R,2S* **‐**cycloxaprid shows that parent compound and metabolite are only found; a similar result is observed with *1S, 2R*‐cycloxaprid. The investigation shows that *1R,2S* **‐**cycloxaprid is conversed into *1S, 2R*‐cycloxaprid. The degradation concentration of *1R, 2S* **‐**cycloxaprid decreases more quickly than the degradation concentration of *1S,2R*‐cycloxaprid (Figure 2). The parent *1R, 2S*‐cycloxaprid is not detected over 96 h, but the *1S, 2R*‐cycloxaprid residue can be still determined at 140 h incubation time. This suggests that the degradation of *1R,2S*‐cycloxaprid decreases more quickly than the *1S, 2R* ‐cycloxaprid. The corresponding degradation kinetics of *1R,2S*‐cycloxaprid and *1S, 2R‐*cycloxaprid are in accord with the pseudo‐first‐order kinetics, and the R^2^ ranged from 0.997 to 0.937 in Puer tea processing. The estimated t_1/2_ values of the *1R, 2S*‐cycloxaprid and *1S, 2R*‐cycloxaprid are 0.97 and 1.1 h, respectively.

The metabolite can be first monitored at 2 h. The metabolite concentration of *1R, 2S*‐cycloxaprid is little than the metabolite of *1S, 2R*‐cycloxaprid. The metabolite of *1R, 2S*‐cycloxaprid increases from 2 h to 48 h, then begin to decrease slowly, but the increase interval is short from 2 h to 24 h with *1S, 2R*‐cycloxaprid.

### 3.4 Stereoselective dissipation of cycloxaprid in Puer tea processing

**FIGURE 3:**
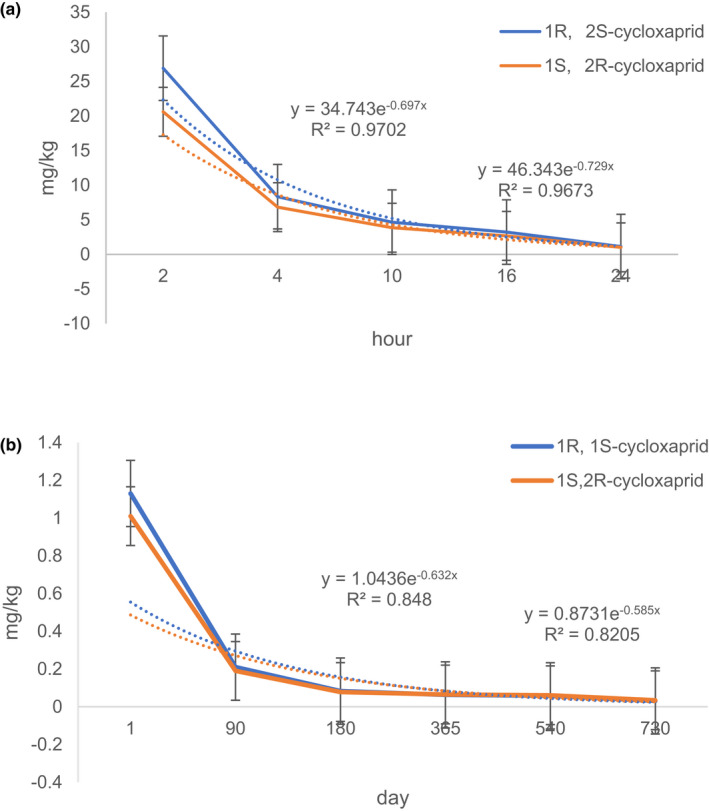
Dissipation kinetics of enantiomerical cyxloxaprid at two divide stages in raw Puer tea: (a) 24 h; (b) 730 days

The fermentation processing with under from several months to several ten years is unique to raw Puer tea. So the degradation of cycloxaprid is still detected over 730 day. The formed reported (Chen et al., 2017) that half‐lives of cycloxaprid in three soils were <24 h. To detect the dissipation of cycloxaprid in Puer tea, the degradation is divided into two stages. The degradation of cycloxaprid decrease more swiftly at 24 incubation time than the stage from one day to 730 days. The corresponding degradation kinetics from 0 (2 h) to 730 day are inconformity to the pseudo‐first‐order kinetics, but the degradation kinetics is good corresponding to divide two curves. The correlation coefficient of cycloxaprid are 0.964 (0.963) and 0.871 (0.852) respectively (Figure 3). To our surprise, the half‐lives of *1R, 2S*‐cycloxaprid and *1S, 2R*‐cycloxaprid are 1.68 h and 1.77 h, but the residue is still detected at 730 days. The reason need be more researched in the future.

**FIGURE 4:**
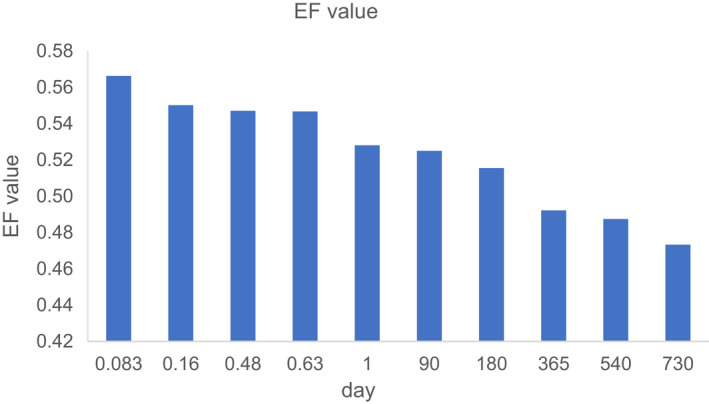
The change of stereoselectivity (EF) in raw Puer tea processing

Stereoselectivity is expressed as EF value. As shown in Figure 4, the beginning of EF value in cycloxaprid is >0.50, and the decrease of EF is obvious from 2 h to 730 days. The result showed that enantioselectivity is significantly observed during raw Puer tea processing. The result is shown that the degradation of cycloxaprid is enantioselectivity under raw Puer tea processing.

### 3.5 Stereoselective dissipation of cycloxaprid in ripen Puer tea processing

**FIGURE 5:**
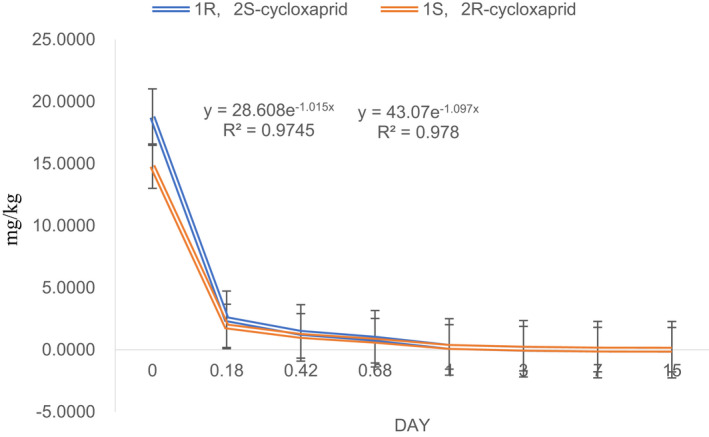
Dissipation kinetics of enantiomerical cycloxaprid during ripen Puer tea processing

Ripen Puer tea is unique processing due to the pile formation at 45 or 60 days. So the degradation of cycloxaprid is detected from starting processing to 45 days. The extractable cyclopraxid fraction quickly decrease at ripen Puer tea processing. The parent cycloxaprid is not detected over 30 days. The corresponding degradation kinetics from 0 (2 h) to 15 days (Figure 5) are conformity to the pseudo‐first‐order kinetics, and the half‐lives of *1R, 2S*‐cycloxaprid and *1S, 2R*‐cycloxaprid are 0.60 days and 0.63 days.

**FIGURE 6:**
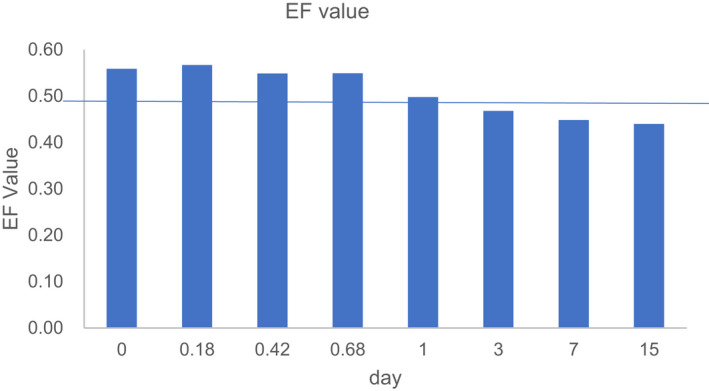
The change of stereoselectivity (EF) in ripen Puer tea processing

The decrease of EF is obvious from 0.56 to 0.44 during ripen Puer tea processing in Figure 6. From the statistical analysis, it showed that there is stereoselective preference for cycloxaprid enantiomers as evidenced as significant difference among the stereoisomers and the racemate in ripen Puer tea processing. The result is shown that the degradation of cycloxaprid is enantioselectivity under raw Puer tea processing.

### 3.6 Dissipation of metabolite in raw Puer tea processing and ripen Puer tea

**FIGURE 7:**
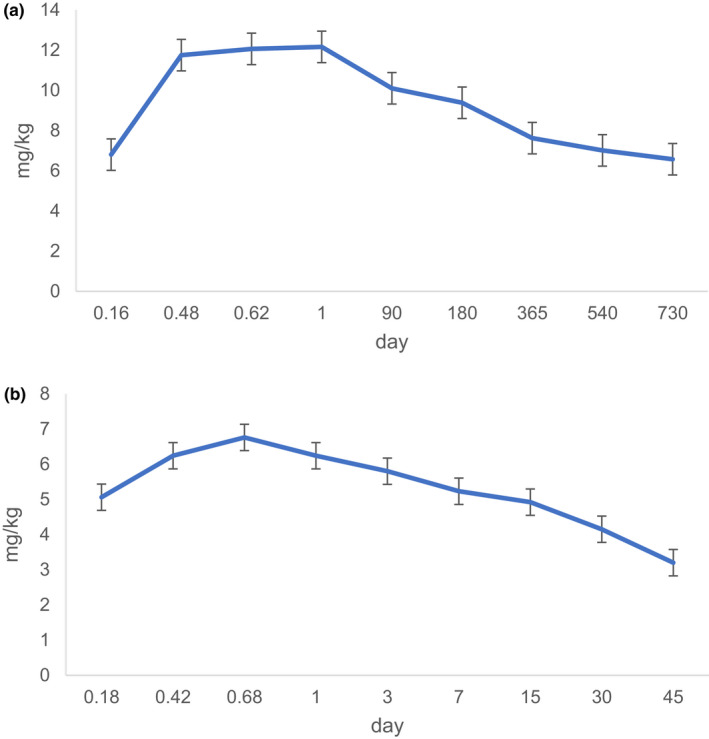
Dissipation kinetics of metabolite in Puer tea processing (a) raw Puer tea (b) ripen Puer tea

When metabolites are produced during Puer tea processing, they are not easy to decompose. So the terminal residue is still detected until 730 days in raw Puer tea processing and 45 days in ripen Puer tea processing (Figure 7). The maximum residue appears at one day (raw Puer tea processing) or earlier (ripen Puer tea processing). The metabolites are higher in residues in raw Puer tea than in ripen Puer tea.

## CONFLICT OF INTEREST

All Authors declare that they have no conflict of interest.

## ETHICAL APPROVAL

This article does not contain any studies with human participants performed by any of the authors.
